# A novel NAP member GhNAP is involved in leaf senescence in *Gossypium hirsutum*

**DOI:** 10.1093/jxb/erv240

**Published:** 2015-05-18

**Authors:** Kai Fan, Noreen Bibi, Susheng Gan, Feng Li, Shuna Yuan, Mi Ni, Ming Wang, Hao Shen, Xuede Wang

**Affiliations:** ^1^Institute of Crop Science, College of Agriculture and Biotechnology, Zhejiang University, Hangzhou 310058, PR China; ^2^Plant Biology Section, School of Integrative Plant Science, Cornell University, Ithaca, NY 14853–5904, USA

**Keywords:** Abscisic acid, GhNAP, *Gossypium hirsutum*, leaf senescence, NAP subfamily, transcription factor.

## Abstract

Premature leaf senescence has a negative influence on the yield and quality of cotton, and several genes have been found to regulate leaf senescence. Howeer, many underlying transcription factors are yet to be identified. In this study, a NAP-like transcription factor (GhNAP) was isolated from *Gossypium hirsutum*. GhNAP has the typical NAC structure and a conserved novel subdomain in its divergent transcription activation region (TAR). GhNAP was demonstrated to be a nuclear protein, and it showed transcriptional activation activity in yeast. Furthermore, the expression of *GhNAP* was closely associated with leaf senescence. GhNAP could rescue the delayed-senescence phenotype of the *atnap* null mutant. Overexpression of *GhNAP* could cause precocious senescence in *Arabidopsis*. However, down-regulation of *GhNAP* delayed leaf senescence in cotton, and affected cotton yield and its fibre quality. Moreover, the expression of *GhNAP* can be induced by abscisic acid (ABA), and the delayed leaf senescence phenotype in GhNAPi plants might be caused by the decreased ABA level and reduced expression level of ABA-responsive genes. All of the results suggested that GhNAP could regulate the leaf senescence via the ABA-mediated pathways and was further related to the yield and quality in cotton.

## Highlight

GhNAP could regulate leaf senescence via the ABA-mediated pathways and is related to the yield and quality of cotton.

## Introduction

Leaf senescence is an accumulative series of physiological and molecular changes that disrupt cellular metabolism (Thomas and Stoddart, 1980; Lim *et al.*, 2007). During leaf senescence, degradation of macromolecules occurs and finally results in its shift from a functionally photosynthetic organ to an actively degenerating and nutrient-recycling tissue (Gan, 2007). Like many other developmental processes, leaf senescence can be regulated by many transcription factors (Guo *et al.*, 2004; Kong *et al.*, 2013). Recently it has been reported that numerous transcription factors, including NAC, WRKY, bZIP, MYB, AP2/EREBP, and C2H2 type zinc finger, are related to leaf senescence (Buchanan Wollaston *et al.*, 2005; Guo and Gan, 2012). In particular, the NAP subfamily (NAC-like, activated by APETALA 3/PISTILLATA) has been genetically and physiologically proved to be an important activator of leaf senescence (Guo and Gan, 2006; Liang *et al.*, 2014).

The NAP members are one of the largest subfamilies of plant-specific NAC (NAM, ATAF1, 2, and CUC2) transcription factors (Duval *et al.*, 2002; Olsen *et al.*, 2005). In the earliest research, the *NAP* gene was found to participate in cell division and expansion of stamens and petals (Sablowski and Meyerowitz, 1998). The NAP subfamily has a highly conserved N-terminal domain (NAC domain) (Aida *et al.*, 1997; Ooka *et al.*, 2003) and a highly divergent C-terminal domain in the transcription activation region (TAR) (Fujita *et al.*, 2004; Tran *et al.*, 2004). The NAP subfamily is very important in various biological functions, such as floral development (Sablowski and Meyerowitz, 1998), root morphogenesis (de Zélicourt *et al.*, 2012), seed development (Meng *et al.*, 2007), and stress responses, including salt (Chen *et al.*, 2014), drought (Meng *et al.*, 2009), and H_2_O_2_ stress (Peng *et al.*, 2009). In addition, the NAP subfamily is also associated with leaf senescence in *Arabidopsis thaliana* (Guo and Gan, 2006), *Oryza sativa* (Liang *et al.*, 2014), *Crocus sativus* (Kalivas *et al.*, 2010), *Bambusa emeiensis* (Chen *et al.*, 2011), and *Triticum aestivum* (Uauy *et al.*, 2006). AtNAP was closely linked to the senescence process of *A. thaliana* rosette leaves, and its corresponding T-DNA insertion knockout lines showed an obvious delay in leaf senescence. In contrast, inducing overexpression of *AtNAP* in the young leaf led to advanced senescence (Guo and Gan, 2006). The NAP subfamily also improves crop yield and quality through regulating leaf senescence. Reduced *OsNAP* expression can delay leaf senescence and increase grain yield in rice (Liang *et al.*, 2014). Furthermore, NAM-B1, an orthologue of AtNAP, was associated with leaf senescence and grain quality in wheat (Uauy *et al.*, 2006).

Cotton is considered as the most important economic crop in the world. However, premature leaf senescence of cotton has occurred frequently in many cotton-growing countries, and it has become one of the main factors restricting cotton yield and quality (Dong *et al.*, 2006). Leaf senescence is mainly determined by the genetic programme in cotton, but its key regulation mechanisms and corresponding transcription factors remain largely unclear. As is known, the NAP subfamily is related to leaf senescence, but there are limited reports about the NAP-like transcription factors in cotton (Meng *et al.*, 2009; Zhang *et al.*, 2011; Huang *et al.*, 2013; Shang *et al.*, 2013). Although some GhNAPs have been isolated from senescing leaf in cotton, it is still unknown whether or not a NAP-like transcription factor in cotton acts as an important factor for triggering cotton leaf senescence (Kong *et al.*, 2013; Shah *et al.*, 2013; Shah *et al.*, 2014). It is also still unknown how a NAP transcription factor plays a crucial part in leaf senescence in cotton.

To explore the possible regulation pathways for extending the green period in cotton, a novel NAP-like transcription factor, GhNAP, was investigated in upland cotton (*Gossypium hirsutum* L.) which may be related to leaf senescence. Due to its rapid responses to leaf senescence signals, GhNAP can be identified as an ideal positive senescence marker in cotton. GhNAP could rescue the delayed-senescence phenotype of the *atnap* null mutant, and overexpression of *GhNAP* could readily cause precocious senescence in *Arabidopsis*. On the other hand, reduction of *GhNAP* expression delayed cotton senescence. Furthermore, GhNAP can mediate abscisic acid (ABA) pathways by regulating several ABA-responsive genes, and the ABA-mediated pathways of GhNAP in senescence may differ from that of AtNAP. In addition, cotton yield and its fibre quality could improve with a reduction of the transcript level of the *GhNAP* gene in cotton.

## Materials and methods

### Plant materials and growth conditions

*Arabidopsis thaliana* seeds of Col-0, *atnap* null mutants (SALK_005010), and all transgenic lines were sown on Petri dishes containing Murashige and Skoog (MS) salts with 0.7% (w/v) phytoagar. After vernalization at 4 °C for 2 d, the dishes were moved to a growth chamber at 22 °C with 60% relative humidity. After 12 d of germination, seedlings were transplanted to pots containing a peat soil:vermiculite:perlite mixture (3:9:0.5, v/v/v). For dark treatment, the fifth leaves were excised and incubated on wet Petri dishes.

*Gossypium hirsutum* L. cv. Zheda B was used in this research. To induce expression of *GhNAP*, 100 μM ABA was applied to 1-month-old wild-type seedlings for 12h. For dark-induced senescence, seedlings were put in a similar environment but in total darkness. GhNAPi homozygous lines, together with the wild-type cotton, were also grown in the experimental field of Zhejiang University. A completely randomized block design was employed. Thereafter, the fourth leaf from the apex was used to measure yellowing at the designated times. The cotton bolls were gathered from the above-measured lines in the harvest season to investigate their yield and quality. Fibre quality traits were examined in the Supervision, Inspection and Test Center of Cotton Quality. Leaves from the GhNAPi and wild-type cotton were detached from 1-month-old plants and placed on wet Petri dishes in continuous darkness.

### Isolation, subcellular localization, and transcriptional activation analysis of GhNAP

*Gossypium hirsutum* expressed sequence tags (ESTs) were downloaded from the NCBI. Through searching and alignment with AtNAP, a unigene was selected to clone the *GhNAP* gene (Pinheiro *et al.*, 2009; de Oliveira *et al.*, 2011). For subcellular localization, the full-length *GhNAP* without the stop codon was amplified, and then inserted into the pCHF3-GFP (green fluorescent protein) vector. Then the pCHF3-GFP and pCHF3-GFP-GhNAP vectors were introduced into *Agrobacterium tumefacines* strain LBA4404, and were transiently expressed in transgenic *Nicotiana benthamiana* plants expressing red fluorescent protein (RFP)–H2B. The transformed leaves were observed by confocal microscopy. For transcriptional activation analysis, the full-length GhNAP, GhNAP-N (amino acids 1–162), and GhNAP-C (amino acids 163–286) were fused in pGBKT7 to construct pGBKT7-GhNAP, pGBKT7-GhNAP-N, and pGBKT7-GhNAP-C, respectively. Yeast strains (Clontech) were transformed with the three resulting constructs and the negative control pGBKT7. The transformants were evaluated on SD/–Trp and SD/–Trp/X-α-Gal/AbA media.

### Evolutionary analysis

For phylogenetic analysis of GhNAP, the amino acid sequences of the NAP subfamily already reported in plants were collected from GenBank. All of the NAP members in this study were aligned using the ClustalX program. Then, MrBayes version 3.1.2 was used to conduct Bayesian analysis (Huelsenbeck and Ronquist, 2001). The conserved motifs among the NAP subfamily were also investigated by the online MEME program (Bailey *et al.*, 2006). The parameters were set as follows: optimum motif width was set to ≥6 and ≤200; maximum number of motifs was set to 15 (Zhao *et al.* 2011). Sequence logos of the conserved NAC domain and a novel subdomain were generated through the WebLogo program (Crooks *et al.*, 2004).

### Physiological measurement and transcript analysis

Chlorophyll, the SPAD value, and membrane ion leakage were measured as previously described (Feibo *et al.*, 1998; Woo *et al.*, 2001). The photosynthetic capacity was determined using an LI-6400 portable photosynthesis system (Hua *et al.*, 2009) and pulse-modulated chlorophyll fluorometer (Chen *et al.*, 2005). The peroxidase (POD) and superoxide dismutase (SOD) activities, and soluble protein and malondialdehyde (MDA) contents were measured using reported methods (Wang *et al.*, 2011). Additionally, the extraction and determination of the endogenous ABA level in the 1-month-old wild-type and GhNAPi cotton was performed as described (Lanoue *et al.*, 2010).

Total RNA was extracted using an RNAprep pure Plant Kit (TIANDZ, China) for cotton and RNAiso Plus (TakaRa) for *Arabidopsis*. The first-strand cDNA was synthesized from DNase-treated RNA with a PrimerScript 1st Strand cDNA synthesis kit (TaKaRa). Quantitative real-time PCR (qRT-PCR) was performed with SYBR premix Extaq (TaKaRa) and the CFX96 Realtime System (BioRad). The primers used for qRT-PCR are listed in Supplementary Table S1 available at *JXB* online. Forty cycles of qRT-PCR were conducted with an annealing temperature of 60 °C. The relative expression levels were calculated by the 2^–ΔΔCt^ method assuming 100% primer efficiency (Schmittgen and Livak, 2008). Three biological replications were performed in all reactions.

### Plasmid construction and plant transformation

For the complementation test, the promoter region of *AtNAP* was amplified from Col-0, and subcloned into the pCHF3 vector to replace the 35S promoter. Then the full-length *GhNAP* was inserted into the ProAtNAP_pCHF3 vector to construct ProAtNAP_pCHF3_GhNAP. For overexpression of *GhNAP*, the open reading frame (ORF) of GhNAP was amplified and inserted into pCHF3. Primers used for constructing different vectors are given in Supplementary Table S2 at *JXB* online. In addition, the pCI-GhNAPi interference vector was generated by cloning the *GhNAP* coding region into the RNA interference (RNAi) expression vector pCI. Then the above three vectors were transferred into *A. tumefacines* strain LBA4404, which was used to transform *A. thaliana* via the floral dip method (Clough and Bent, 1998). Putative transgenic plants were selected on MS medium containing 50mg l^–1^ kanamycin. The antibiotic-resistant T_1_ transgenic lines were selected, and were further verified by PCR and RT-PCR; phenotypic analyses were performed in the T_2_ generation and were further confirmed in the T_3_ generation. Furthermore, *G. hirsutum* L. cv. Zheda B was transformed with the pCI-GhNAPi vector according to the method of Luo and Wu (1988). The plants were first screened by 500mg l^–1^ kanamycin. Then the positive plants were further confirmed by PCR and RT-PCR. Selected transgenic plants were analysed by Southern blotting using the DIG High Prime DNA Labeling and Detection Strater Kit I (Roche). Homozygous plants were used in all experiments.

### Isolation of *GhNAP* and *GhSAG113* promoters

Total genomic DNA was extracted from cotton leaf. The Genome Walking Kit (TaKaRa) was used to clone the promoter region of *GhNAP* and *GhSAG113*. The gene-specific primers were designed based on the known sequences (Supplementary Table S3 at *JXB* online), and the specific PCR products were cloned and sequenced. The GhNAP promoter sequence was then searched in the PLACE database to investigate the putative *cis*-elements.

### Yeast one-hybrid assay

The yeast one-hybrid (Y1H) assay was performed using the Matchmaker^®^ Gold Yeast One-Hybrid Library Screening System (Clontech) according to the manufacturer’s instructions. The promoter of *GhSAG113* and the ORF of GhNAP were ligated into pAbAi and pGADT7 vectors, respectively, to generate the pAbAi-GhSAG113 and pGADT7-GhNAP vectors, respectively. The pAbAi-GhSAG113 plasmid was linearized and transformed into the Y1H Gold strain. Positive yeast cells were selected on SD/–Ura medium, and then transformed with pGADT7-GhNAP. The transformants were obtained on SD/–Leu medium, and were evaluated on SD/–Leu/AbA plates.

### Statistical analysis

Data were subjected to PROC GLM using the SAS version 8.0 statistical software designed by the SAS Institute (Cary, NC, USA). The significant differences among different groups were evaluated by least significant difference (LSD) multiple range tests (*P*<0.05).

## Results

### Isolation and molecular characterization of GhNAP in *G. hirsutum*

**Fig. 1.**
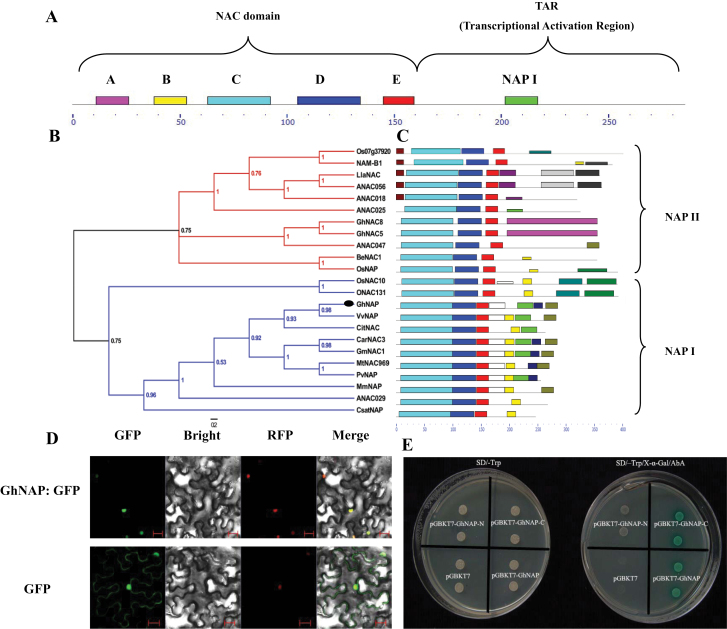
Structural, phylogenetic, subcellular localization, and transcriptional activation analysis of GhNAP. (A) The NAC domain and TAR of GhNAP. Subdomains are shown by rectangles. (B) Phylogenetic analysis of GhNAP. The phylogenetic tree was constructed using the Bayesian method based on the multiple alignments of 23 NAP protein sequences. The tree is unrooted. The numbers in the clades are posterior probability values. (C) Distribution of 15 putative conserved motifs in the NAP subfamily by the MEME search tool. Different motifs are represented by various boxes. The location of each motif can be estimated using the scale at the bottom. The groups within the NAP subfamily are classified by different brackets according to the phylogenetic relationship. (D) Subcellular localization of GhNAP. The GhNAP–GFP fusion protein and free GFP were transiently expressed in transgenic *Nicotiana benthamiana* plants expressing RFP–H2B, and the transformed leaves were observed by confocal microscopy. Images in the first column show cells with the GFP signal. Images in the second column show the bright-field view of the same cells. Images in the third column show the same cells with the RFP signal, and the images in the fourth column are the overlays of the bright-field and fluorescent images. Scale bar=20 μm. (E) Transcriptional activation analysis of GhNAP in yeast. The full length and the N- (GhNAP-N) and C-terminal (GhNAP-C) regions of GhNAP were inserted into the pGBKT7 vector. The pGBKT7 plasmid was used as the negative control. The above four constructs were transformed into yeast on SD/–Trp and SD/–Trp/X-α-Gal/AbA media for examination of growth. (This figure is available in colour at *JXB* online.)

AtNAP was used as the query to search *G. hirsutum* ESTs. Through comprehensive analysis with AtNAP, a unigene (EV490808, DR455718, DN800623, and CA992692) was selected and tentatively called GhNAP. GhNAP contained the whole ORF, so a pair of PCR primers was designed to clone the GhNAP sequence. Sequencing results showed that GhNAP contained the 861bp ORF encoding 286 amino acids (Fig. 1A).

To understand the evolutionary history of GhNAP, a phylogenetic tree including some reported NAP proteins and GhNAP was constructed. Through evolutionary analysis, it was found that the NAP subfamily could be further divided into two groups (NAPI and NAPII) (Fig. 1B). The NAPI group contained 11 transcription factors, while the NAPII group had 12 NAP members. Interestingly, both GhNAP and AtNAP belonged to the NAPI group. Furthermore, the motif distribution was consistent with the classification of the phylogenetic analysis (Fig. 1C). Further analysis of the conserved domain revealed that GhNAP shared a high percentage identity (91%) with AtNAP. Therefore, it can be revealed that GhNAP may be a homologue of AtNAP.

Sequence alignment among NAP members revealed that the NAC domain of GhNAP was highly conserved and could be further divided into five typical subdomains. In addition, a novel subdomain in its TAR was relatively conserved within the NAPI group (Supplementary Fig. S1 at *JXB* online). It was therefore tentatively called subdomain NAPI. Furthermore, the DNA-binding domain (DBD domain) in subdomain C and the nuclear localization signal (NLS) in subdomain D were identified in GhNAP and highly conserved with other NAP members.

### Subcellular localization of GhNAP

To determine the subcellular localization of GhNAP, the GhNAP–GFP fusion vector was transiently expressed in RFP–H2B transgenic *N. benthamiana*. The green fluorescent signal from GhNAP–GFP expression was observed exclusively in the nucleus, which was confirmed with the red fluorescent signal from RFP–H2B, a fusion protein often used to visualize the chromosomal architecture in cells (Fig. 1D). However, the free GFP signal was distributed throughout the cell.

### Transcriptional activation activity of GhNAP

To examine whether GhNAP has transcriptional activation activity, the N- and C-terminal fragments as well as the full-length GhNAP were fused to the GAL4 DBD of the pGBKT7 vector. The resulting constructs and the negative vector control pGBKT7 were transformed into a yeast strain. All of the transformants grew well on SD/–Trp medium, while only the cells containing the pGBKT7-GhNAP and pGBKT7-GhNAP-C plasmid could grow and simultaneously turned blue on SD/–Trp/X-α-Gal/AbA medium (Fig. 1E).

### Expression profile of *GhNAP* during leaf senescence in cotton

**Fig. 2.**
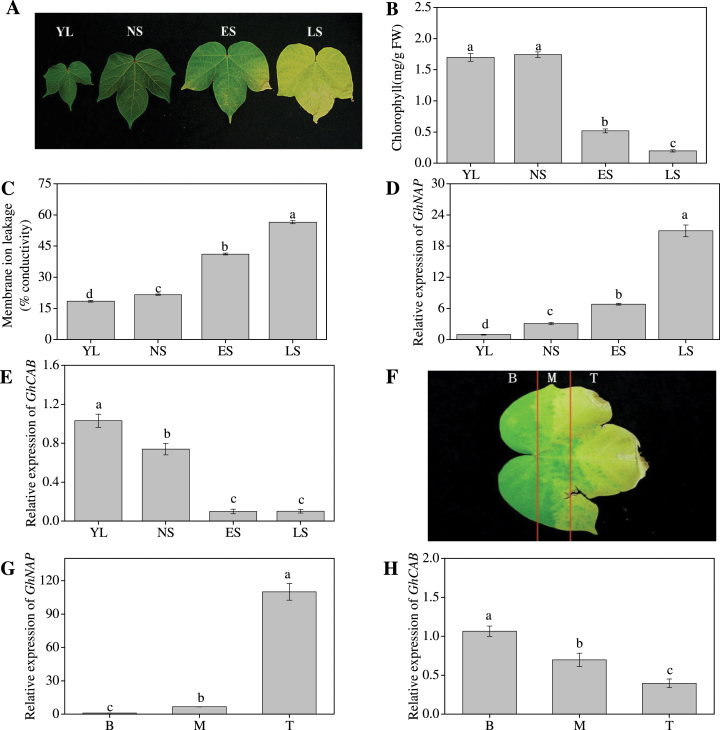
Physiological and molecular analysis of *GhNAP* expression during leaf senescence in cotton. (A–E) Cotton leaves at various developmental stages and *GhNAP* expression. YL, a young leaf half the size of a fully expanded leaf; NS, a fully expanded, non-senescent leaf; ES, an early senescent leaf, with <50% leaf area yellowing; LS, a late senescent leaf, with >50% leaf area yellowing. Chlorophyll content (B), membrane ion leakage (C), and relative expression of *GhNAP* (D) and *GhCAB* (E) at the corresponding stage of (A). Error bars indicate the standard error (*n*=3). Significant differences between means are represented by different letters. A similar statistical analysis was conducted in the following parts. (F–H) Different parts in the senescing cotton leaf and the relative expression level of *GhNAP* and *GhCAB*. B, base; M, middle; T, tip. *EF1α* was used as the standard control in all qRT-PCR experiments in cotton. (This figure is available in colour at *JXB* online.)

The expression of *GhNAP* was examined in young leaves (YL), non-senescent leaves (NS), early senescent leaves (ES), and late senescent leaves (LS) (Fig. 2A). Consistent with the leaf phenotype, the chlorophyll loss and membrane ion leakage of ES and LS were higher than those of YL and NS (Fig. 2B, C). Moreover, compared with YL and NL, the expression of *GhNAP* was higher in ES and LS, whereas *GhCAB*, a negative senescence marker gene, was expressed at a lower level in the same leaves (Fig. 2D, E). During the senescence process (Fig. 2F), more *GhNAP* expression was detected in the yellow tip (T) than in other parts (Fig. 2G). The present findings were further strengthened by the opposite expression level of *GhCAB* (Fig. 2H). Furthermore, YL were treated in a dark environment for 7 d. The longer the dark treatment was, the greater was the chlorophyll loss and membrane ion leakage (Supplementary Fig. S2A, B at *JXB* online). These results were in line with the decrease in the expression of *GhCAB* (Supplementary Fig. S2C) and the increase in *GhNAP* expression (Supplementary Fig. S2D).

### Complementation of *Arabidopsis atnap* null mutants with *GhNAP*

**Fig. 3.**
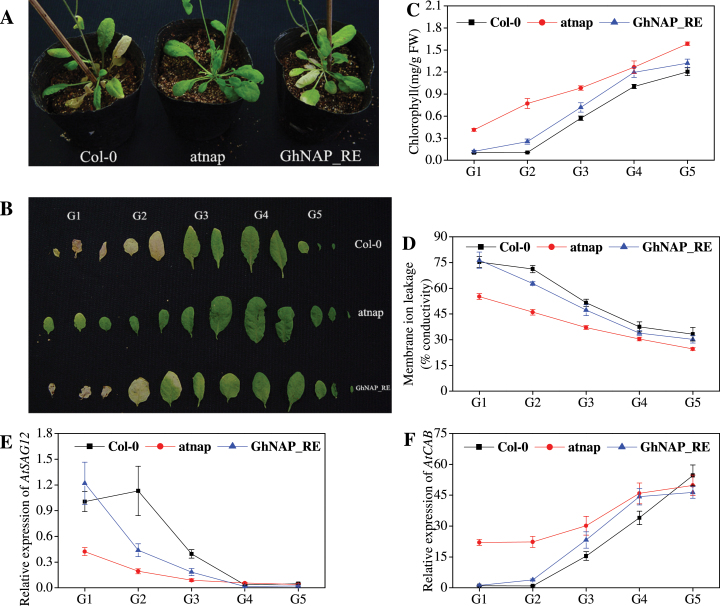
Natural senescence of GhNAP-complemented lines. (A, B) Phenotypes of Col-0 and GhNAP_RE lines under a normal environment after 60 d growth. G1–G5, five groups of detached leaves according to the senescent condition. (C–F) Chlorophyll content (C), membrane ion leakage (D), and relative expression of *AtSAG12* (E) and *AtCAB* (F) in the detached leaves of the five groups. *AtActin2* was used as the standard control in all of the qRT-PCR experiments in *Arabidopsis*. (This figure is available in colour at *JXB* online.)

Previous reports have shown that the *atnap* null mutant can delay leaf senescence (Guo and Gan, 2006). To test whether GhNAP is a functional homologue of AtNAP, the GhNAP ORF, driven by the promoter region of *AtNAP*, was transformed into the *atnap* null mutant (Supplementary Fig. S3 at *JXB* online). The leaves from the GhNAP-complemented lines (GhNAP _RE) senesced in a similar pattern to the leaves from Col-0, but senesced much more quickly than the leaves from *atnap*, both phenotypically (Fig. 3A, B) and in terms of chlorophyll content (Fig. 3C), membrane ion leakage (Fig. 3D), and relative expression of *AtSAG12* (Fig. 3E) and *AtCAB* (Fig. 3F). After dark treatment for 5 d, detached leaves of GhNAP _RE exhibited wild-type-like yellowing (Supplementary Fig. S4).

### Ectopic expression of *GhNAP* in *Arabidopsis*

**Fig. 4.**
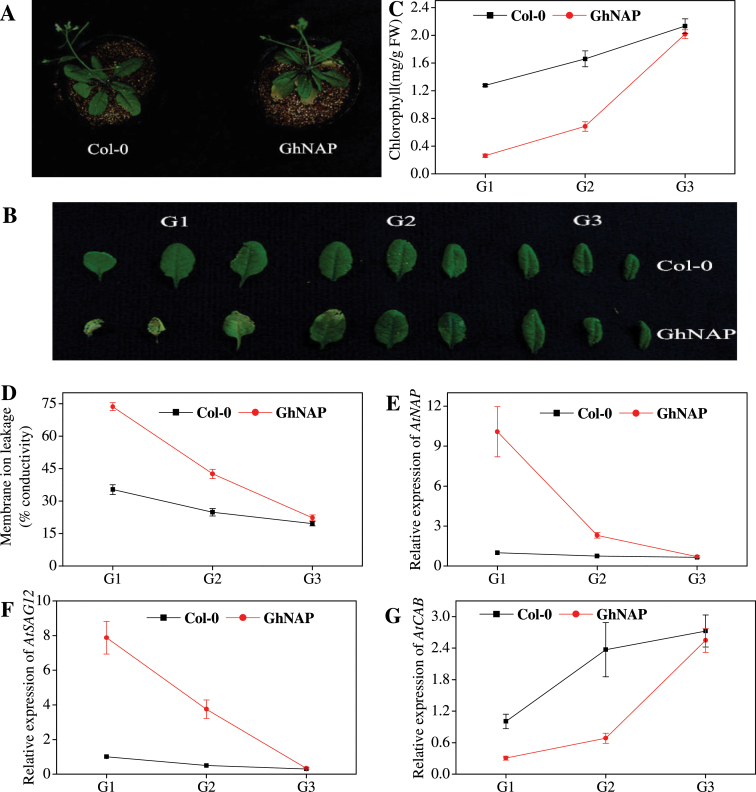
Natural senescence process of *Arabidopsis* leaves in Col-0 and GhNAP lines. (A, B) Phenotypes of Col-0 and GhNAP lines under a normal environment after 30 d growth. G1–G3, three groups of detached leaves according to the senescent condition. (C–G) Chlorophyll content (C), membrane ion leakage (D), and relative expression of *AtNAP* (E), *AtSAG12* (F), and *AtCAB* (G) in the detached leaves of the three groups. (This figure is available in colour at *JXB* online.)

The GhNAP coding sequence, driven by the 35S promoter, was introduced into the wild-type Col-0 to overexpress *GhNAP* (Supplementary Fig. S3 at *JXB* online). After 20 d growth, the GhNAP overexpressors displayed the senescence phenotype, while the no yellowing phenotype was found in Col-0. After 30 d, the detached leaves were divided into three groups (G1–G3) (Fig. 4A, B). Compared with Col-0, the GhNAP lines showed much lower chlorophyll contents in the G1 and G2 leaves (Fig. 4C), while the GhNAP lines had much higher membrane ion leakage (Fig. 4D). The relative expression level of *AtNAP*, *AtSAG12*, and *AtCAB* also reflected the similar senescing process (Fig. 4E–G). Furthermore, compared with the Col-0 leaves, the GhNAP leaves showed the precocious senescence phenotype after 3 d in darkness (Supplementary Fig. S5).

**Fig. 5.**
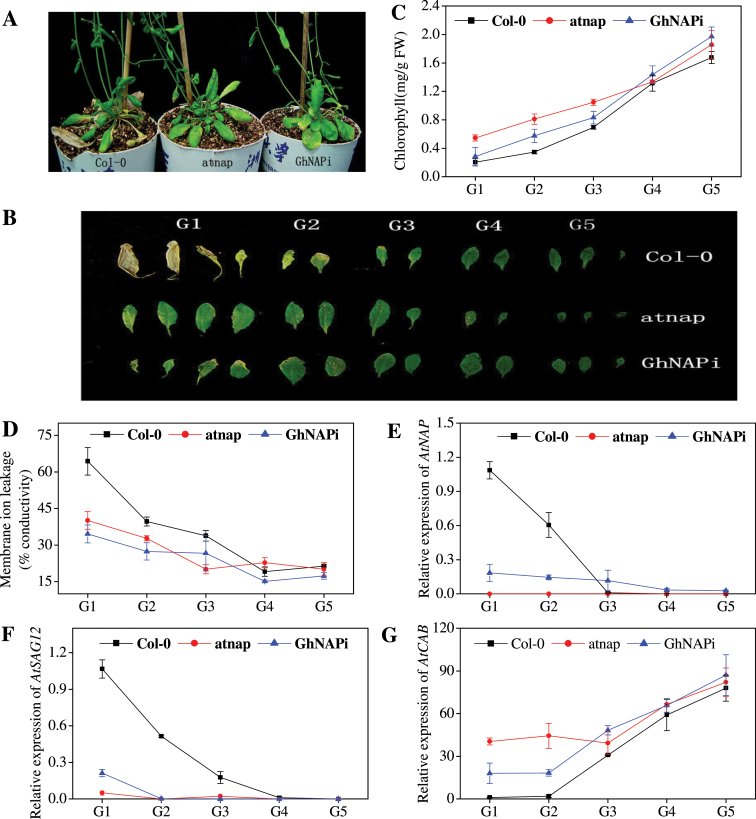
Natural senescence process of *Arabidopsis* leaves in Col-0, *atnap*, and GhNAPi lines. (A, B) Phenotypes of Col-0, *atnap*, and GhNAPi lines under a normal environment after 50 d growth. G1–G5, ﬁve groups of detached leaves according to the senescent condition. (C–G) Chlorophyll content (C), membrane ion leakage (D), and relative expression of *AtNAP* (E), *AtSAG12* (F), and *AtCAB* (G) in the detached leaves of the five groups. (This figure is available in colour at *JXB* online.)

In addition, the GhNAP interference vector (pCI-GhNAPi) was transformed into Col-0 (Supplementary Fig. S3 at *JXB* online). After 35 d growth, leaf senescence occurred first in Col-0. Subsequently, GhNAPi leaves started yellowing after 40 d. After 50 d, leaf senescence was seen in all lines, and detached leaves were divided into five groups (G1–G5) (Fig. 5A, B). The chlorophyll loss and membrane ion leakage in the G1 and G2 leaves of GhNAPi were significantly lower than in the counterpart leaves of Col-0, but higher than those of *atnap* (Fig. 5C, D). A similar ageing process was also shown by the relative expression level of *AtNAP*, *AtSAG12*, and *AtCAB* (Fig. 5E–G). Then the fifth rosette leaves of three lines were incubated in the dark for 5 d (Supplementary Fig. S6). All of these leaves exhibited the senescent phenotype, but the highest level of leaf senescence was observed in Col-0, followed by GhNAPi, and then *atnap*.

### Delayed-senescence phenotype through down-regulation of *GhNAP* in cotton

**Fig. 6.**
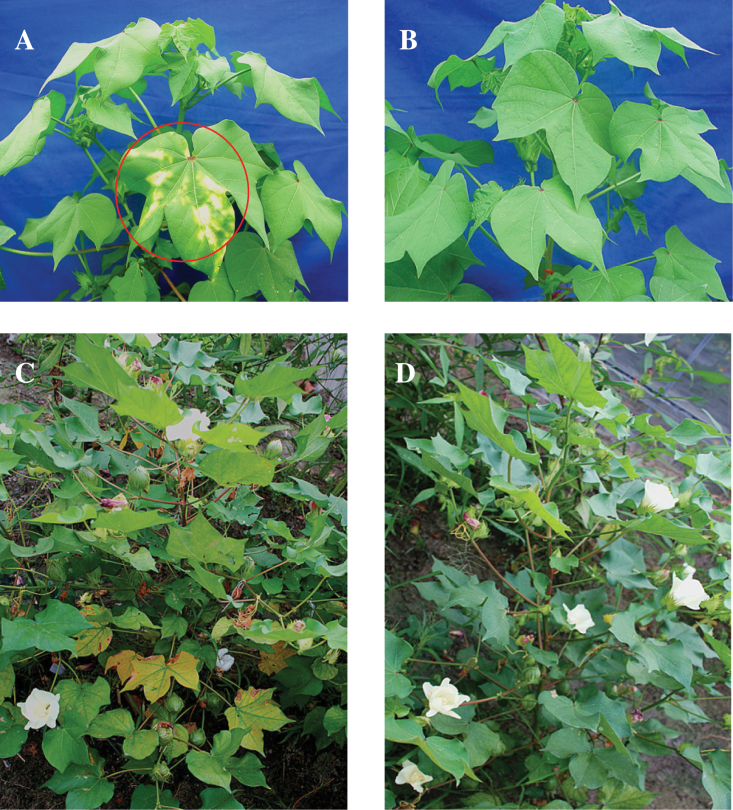
Phenotypes of the GhNAPi and wild-type cotton. (A and C) Wild-type cotton. (B and D) GhNAPi cotton. (A) and (B) were photographed at 60 days after planting (DAP). The circle indicates the non-transgenic seedling leaf with the withered dot after screening with 500mg l^–1^ kanamycin. (C) and (D) were photographed at 120 DAP. (This figure is available in colour at *JXB* online.)

To confirm GhNAP’s function further, the GhNAPi transgenic lines were selected through transformation of the pCI-GhNAPi vector (Supplementary Fig. S7 at *JXB* online). Then the GhNAPi and corresponding wild-type lines were grown in the field. The GhNAPi lines showed delayed senescence especially during the later stage of growth (Fig. 6).

**Fig. 7.**
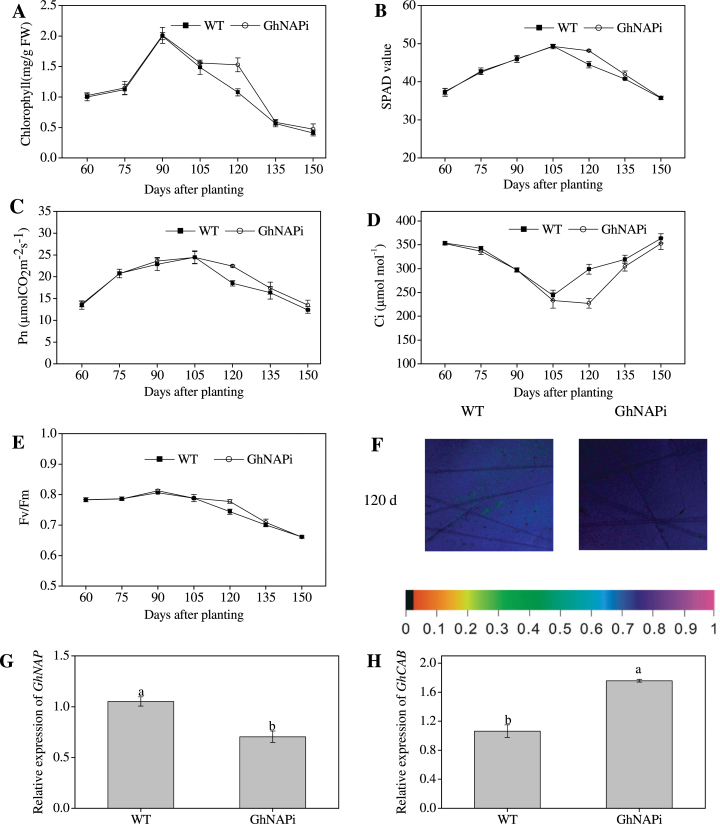
Physiological and molecular analysis of cotton leaf in wild-type (WT) and GhNAPi lines. (A–E) Chlorophyll content (A), SPAD value (B), net photosynthetic rate (P_n_; C), intercellular CO_2_ concentration (C_i_; D), and *F*_v_/*F*_m_ ratio (E) of cotton leaf from 60 to 150 DAP. (F) Emission spectra image on *F*_v_/*F*_m_ of the corresponding leaf at 120 DAP. The bar on the bottom shows the *F*_v_/*F*_m_ value. (G, H) Relative expression of *GhNAP* and *GhCAB* at 120 DAP. (This figure is available in colour at *JXB* online.)

As the striking phenotypic change, leaf yellowing was measured to investigate the leaf senescence every 15 d from 60 days after planting (DAP) to 150 DAP across the main growth period of cotton. At 120 DAP, leaves of GhNAPi had a much higher chlorophyll content and SPAD value than those of the wild type (Fig. 7A, B). The net photosynthetic rate (P_n_) and the *F*_*v*_/*F*_m_ ratio increased at first and gradually declined after 105 DAP, but at 120 DAP the decline of P_n_ and the *F*_v_/*F*_m_ ratio was significantly slower in GhNAPi than that in the wild type (Fig. 7C, E, F). However, the intercellular CO_2_ concentration (C_i_) showed the opposite effects (Fig. 7D). Moreover, *GhNAP* expression was significantly lower at 120 DAP in GhNAPi than in the wild type (Fig. 7G), but *GhCAB* showed the opposite tendency (Fig. 7H). The MDA content, soluble protein content, SOD activity, and POD activity showed a significant difference at 120 DAP between the wild type and GhNAPi (Supplementary Fig. S8 at *JXB* online). In addition, the detached leaves were induced by being kept in the dark for 3 d (Supplementayr Fig. S9A, B). Higher chlorophyll content and lower membrane ion leakage were measured in GhNAPi than in the wild type (Supplementary Fig. S9C, D). The relative expression level of *GhNAP* and *GhCAB* showed similar results (Supplementary Fig. S9E, F).

### Effect on cotton yield and quality through reduced expression of *GhNAP*

The yield and quality of the GhNAPi line were evaluated in the field. There was no significant statistical difference in agronomic traits (plant height, fruit branches, and nodes) and some yield components (boll numbers, seed cotton yield, and boll weight) (Table 1). However, the lint yield of GhNAPi (32.59g plant^–1^) was higher than that of the wild type (28.19g plant^–1^). The percentage lint was 38.08% for the wild type, while it is 41.79% for the GhNAPi cotton. Compared with the wild-type cotton, the lint yield and percentage of GhNAPi were increased by >15% and 9%, respectively. Furthermore, fibre quality, especially fibre length, was significantly different between the GhNAPi and wild-type lines (Table 2). Specifically, the GhNAPi cotton produced fibre with significantly shorter length (27.03mm) than wild-type cotton (29.10mm). Compared with the wild type, the fibre length of GhNAPi was reduced by >7%.

**Table 1.** Comparison of yield components between wild-type and GhNAPi lines

Cotton type	Plant height (cm)	Fruit branches (no. per plant)	Fruit nodes (no. per plant)	No. of bolls per plant	Seed cotton yield (g plant^–1^)	Lint yield (g plant^–1^)	Boll weight (g)	Percentage lint
Wild type	115.08±11.92 a	16.79±1.00 a	53.79±6.69 a	18.00±1.00 a	74.07±3.42 a	28.19±0.90 b	4.12±0.06 a	38.08±0.65 b
GhNAPi	107.75±7.51a	17.13±1.19a	63.46±0.97a	19.67±1.53 a	78.00±2.36 a	32.59±0.81 a	3.98±0.20 a	41.79±0.24 a

Values in each column followed by different letters are significantly different at *P*<0.05.

**Table 2.** Comparison of quality traits between wild-type and GhNAPi lines

Cotton type	Fibre length (mm)	Uniformity (%)	Micronaire value	Elongation rate (%)	Strength (cN/tex)
Wild type	29.10±0.08 a	85.97±0.80 a	5.13±0.09 b	6.40±0.00 b	28.30±0.35 a
GhNAPi	27.03±0.50 b	83.67±0.67 a	5.33±0.12 a	6.60±0.00 a	28.43±0.35 a

Values in each column followed by different letters are significantly different at *P*<0.05.

### Relationship between GhNAP and ABA responses

**Fig. 8.**
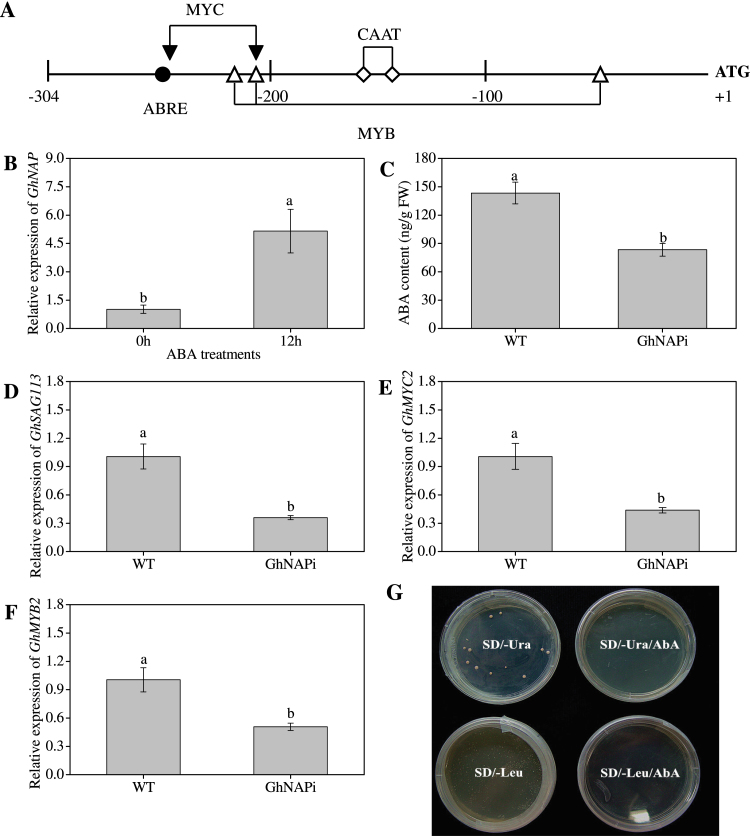
Relationship between GhNAP and ABA pathways. (A) Distribution of *cis*-elements in the promoter region of *GhNAP*. The main *cis*-elements are indicated as follows: filled circles, ABRE; filled inverted triangles, MYC recognition site; open triangles, MYB recognition site; open diamonds, CAAT. (B) Effects of ABA on GhNAP expression in cotton. (C) Endogenous levels of ABA in WT and GhNAPi lines. (D–G) Expression of ABA-related genes in wild-type and GhNAPi plants. (G) Interaction between GhNAP and the promoter of *GhSAG113* by Y1H assay. (This figure is available in colour at *JXB* online.)

To elucidate the possible regulatory mechanism, some putative *cis*-elements were identified in the promoter region of *GhNAP* (Fig. 8A). Surprisingly, not only the common *cis*-element (CAAT-box), but also several specific *cis*-elements related to the ABA responses (ABRE, and recognition sites for MYB and MYC) existed in the promoter region of *GhNAP*. In addition, qRT-PCR analysis showed that the expression pattern of *GhNAP* was highly increased after ABA treatment (Fig. 8B). To explore further the function of GhNAP in the ABA responses, the endogenous ABA level was measured in GhNAPi and wild-type leaves. The results showed that the ABA content was significantly lower in GhNAPi than in wild-type leaves (Fig. 8C). Furthermore, the expression levels of ABA-related genes were analysed in GhNAPi and wild-type lines. Compared with the wild-type lines, *GhSAG113*, *GhMYC2*, and *GhMYB2* exhibited remarkably decreased expression levels in the GhNAPi plants (Fig. 8D–F). In particular, the expression level of *GhSAG113* was reduced >70% in GhNAPi plants. However, the other four genes did not show a significant difference between GhNAPi and wild-type plants (Supplementary Fig. S10 at *JXB* online). Then, the Y1H assay was performed to investigate the interaction between GhNAP and *GhSAG113* (Fig. 8D). The promoter regions of *GhSAG113* (1000bp) were isolated and introduced into the genome of the Y1H Gold strain as the bait reporter strain. Subsequently, the pGADT7-GhNAP vector was transformed into the bait reporter strain. The transformants exhibited normal growth on SD/–Leu medium, but could not grow on SD/–Leu/AbA medium.

## Discussion

### GhNAP is a novel NAP member in *Gossypium hirsutum*

The NAP subfamily are plant-specific transcriptional factors, and are related to plant growth and development, stress responses, and leaf senescence (Sablowski and Meyerowitz, 1998; Guo and Gan, 2006; Uauy *et al.*, 2006). Numerous NAPs have been isolated from different plant species (Meng *et al.*, 2009; Chen *et al.*, 2011; Fan *et al.*, 2014). However, as an important cash crop, cotton lacks systematic research on the NAP subfamily. Thus, studies were focused on cotton in order to find a novel NAP member in *G. hirsutum* which can regulate leaf senescence.

In the current study, a novel transcription factor GhNAP was successfully identified in *G. hirsutum* (Fig. 1A). Consistent with other NAP proteins (Meng *et al.*, 2007; Kalivas *et al.*, 2010), GhNAP contained a conserved NAC domain and a divergent TAR (Fig. 1A; Supplementary Fig. S1 at *JXB* online). Furthermore, the NAC domain can be further divided into five highly conserved subdomains named A–E. The NAC domain, in which WKATGXD was located, mainly acted in DNA binding, while the TAR may determine its specific functions (Ooka *et al.*, 2003). The putative NLS in subdomain D suggested its nuclear localization. Similar to other NAPs (Guo and Gan, 2006; Chen *et al.*, 2014), transient expression in tobacco confirmed that GhNAP was a nuclear protein (Fig. 1D). In addition, transcriptional activation analysis demonstrated tha GhNAP and its C-terminus had transcriptional activation activity in yeast (Fig. 1E). These results indicated that GhNAP may function as a transcription factor in cotton. Through the phylogenetic analysis of the NAP subfamily in the plant kingdom, it was shown that the NAP subfamily might consist of two groups, namely NAPI and NAPII (Fig. 1B, C). GhNAP belonged to the NAPI group, and clustered with AtNAP. A complementation test indicated that GhNAP could restore the *Arabidopsis atnap* null mutant phenotype to the normal wild-type phenotype (Fig. 3). These results collectively indicated that GhNAP is a homologue of AtNAP in cotton. Moreover, GhNAP and other NAP members had a relatively conserved domain in the divergent TAR. The specific subdomain called subdomain NAPI may be specific for NAPI members, indicating certain functions shared by the NAPI group.

### GhNAP is a senescence regulator during leaf development

The NAP subfamily act as a transcription factors to regulate leaf senescence in many plants (Guo and Gan, 2006; Chen *et al.*, 2011; Liang *et al.*, 2014). In this study, GhNAP played an important part in leaf senescence in cotton. First, the transcriptional analysis of *GhNAP* expression from leaves of different ages showed that *GhNAP* was highly expressed not only in the yellow leaf region, but also in the yellowing part (Fig. 2). Previous studies had reported that the NAC family had a similar expression pattern between natural and dark-induced senescence (Lin and Wu, 2004; Buchanan Wollaston *et al.*, 2005; Fukao *et al.*, 2012). Therefore, leaf senescence was induced by darkness in this study. With the extension of the amount of time in the dark period, leaf senescence gradually became more severe and the expression level of *GhNAP* increased correspondingly (Supplementary Fig. S2 at *JXB* online). This result indicated that *GhNAP* was abundantly expressed under dark-incubated leaf senescence.

As a homologue of AtNAP in cotton, GhNAP could restore the delayed leaf senescence phenotype in the *atnap* null mutant to a normal wild-type phenotype during natural or dark-induced leaf senescence (Fig. 3; Supplementary Fig. S4 at *JXB* online). Overexpression of GhNAP in *Arabidopsis* caused precocious leaf senescence, and plants exhibited an increase in chlorophyll loss, membrane ion leakage, and the expression level of *AtSAG12* and *AtNAP*, with a decline in the expression level of *AtCAB* (Fig. 4). After 3 d in darkness, yellowing was much more serious in GhNAP-overexpressing than in wild-type lines (Supplementary Fig. S5 at *JXB* online). In addition, the GhNAPi cassette (GhNAP interference vector) was ectopically transformed in *A. thaliana*. The leaf senescence of the GhNAPi transgenic mutant was intermediate between that of the wild type and the *atnap* null mutant (Fig. 5). The GhNAPi line also showed delayed leaf senescence under a dark environment (Supplementary Fig. S6).

Through the interference with *GhNAP* expression in cotton, the GhNAPi transgenic cotton showed an obvious delay in leaf senescence especially at the later stage of growth (Fig. 6). The physiological parameters also reflected the similar trend of leaf senescence in GhNAPi lines. In particular, at 120 DAP, the GhNAPi leaves contained a higher chlorophyll content and photosynthetic properties than the corresponding leaves of the wild type, indicating that the reduced expression of *GhNAP* extended the leaf functional period (Fig. 7A–F; Supplementary Fig. S8 at *JXB* online). Furthermore, *GhNAP* was expressed at a much lower level at 120 DAP in GhNAPi than in wild-type plants (Fig. 7G), whereas GhNAPi showed a higher transcript profile of *GhCAB* (Fig. 7H). Moreover, GhNAPi lines also showed a delay in dark-induced leaf senescence (Fig. S9).

Due to its rapid responses to leaf senescence signals and the sustained high expression patterns during natural and dark-induced leaf senescence, *GhNAP* can be regarded as an ideal marker to demonstrate leaf senescence in cotton. Taken together, these results suggested that GhNAP played crucial roles in leaf senescence in cotton under both a natural and a dark environment.

### GhNAP influences yield and quality in cotton through regulation of leaf senescence

Although leaf senescence is an evolutionarily selected developmental process (Buchanan Wollaston *et al.*, 2003), it may negatively affect crop yield and quality by limiting the growth phase (Lim *et al.*, 2007). Due to the delayed-senescence phenotype in GhNAPi, the lint yield significantly increased, which led further to a percentage gain in lint (Table 1). However, the fibre length declined sharply in the GhNAPi line (Table 2). The decline of fibre length was probably associated with the inhibition of cell elongation. As was previously reported (Sablowski and Meyerowitz, 1998), partial loss of GhNAP function perhaps inhibited cell elongation, and subsequently affected the fibre length. One probable explanation may be that a pulse of *GhNAP* expression is required for cell expansion. Furthermore, some MYB transcription factors were related to cell expansion in cotton (Walford *et al.*, 2011), and the promoter region of *GhNAP* contained at least three recognition sites for MYB (Fig. 8A). Thus, these results further confirmed the explanation that a definite level of *NAP* expression may be necessary for proper cell elongation. In addition, the higher lint yield and lint percentage in this study may be related to the thickened secondary cell wall in GhNAPi fibre. In previous reports, many MYB members have been shown to regulate secondary cell wall biosynthesis and deposition (Zhong *et al.*, 2007; Sun *et al.*, 2015). Therefore GhNAP might regulate secondary cell wall biosynthesis and deposition, which has been confirmed in other NAC members (Zhong *et al.*, 2006). Furthermore, more fibre cells in GhNAPi probably resulted in the higher lint yield and lint percentage. The endogenous ABA level was significantly lower in GhNAPi than in wild-type lines (Fig. 8C), and ABA is inhibitory to fibre development (Kim and Triplett, 2001). Hence, GhNAPi lines might have more fibre cells through ABA regulation. However, further investigations are still needed to prove these hypotheses.

### GhNAP regulates different ABA pathways during leaf senescence

ABA is one of the plant hormones that can promote leaf senescence (Zhang, 2014). In this study, GhNAP could be highly induced under ABA treatment (Fig. 8B). Furthermore, various *cis*-regulatory elements, including ABRE, and recognition sites for MYB and MYC, were identified in the promoter region of *GhNAP* (Fig. 8A). These *cis*-elements and their respective transcription factors are very important in ABA responses (Abe *et al.*, 1997). These results suggested a close relationship between GhNAP and ABA pathways in leaf senescence. In addition, the interference with *GhNAP* expression in cotton led to delayed senescence under a natural or dark environment (Figs 6, 7; Supplementary Figs S8, S9 at *JXB* online), which was related to the reduction of endogenous ABA content and the decreased expression levels of some ABA-related genes (Fig. 8C–F). All of these results indicated that the delayed yellowing in natural or dark-induced senescence in the GhNAPi transgenic lines might result from the reduced endogenous ABA levels. Hence, GhNAP may regulate leaf senescence through ABA-mediated pathways.

In *Arabidopsis*, the direct target of AtNAP was reported to be *SAG113* (Zhang and Gan, 2012). The unique ABA–AtNAP–SAG113 regulatory chain can control ABA-regulated stomatal movement and water loss specifically during leaf senescence. In this study, qRT-PCR results showed that the expression level of *GhSAG113* (the homologue of *SAG113*) was reduced >70% in the GhNAPi lines (Fig. 8D). In addition, GhNAP is the homologue of AtNAP. Therefore, the interaction between GhNAP and *GhSAG113* was investigated (Fig. 8G). The Y1H assay showed that GhNAP did not bind directly to the promoters of *GhSAG113*, indicating that GhNAP interaction proteins may bind to the promoters of *GhSAG113*. Thus, the regulation model between GhNAP and ABA in cotton differs from the ABA–AtNAP–SAG113 regulatory chain in *Arabidopsis* during leaf senescence.

Overall, the results showed that a novel NAP member (GhNAP) in cotton was closely associated with leaf senescence via ABA-mediated pathways. Down-regulation of *GhNAP* in cotton could delay leaf senescence, and affected the yield and fibre quality. The findings presented here open up a new avenue forresearchers to investigate further the structure and function of the NAP subfamily in plants, and provided a candidate gene for plant breeding.

## Supplementary data

Supplementary data are available at *JXB* online.

Figure S1. The conserved domain of GhNAP protein.

Figure S2. Physiological and expression analysis of *GhNAP* in cotton leaves during extended darkness.

Figure S3. Molecular analysis of the GhNAP_RE, GhNAP, and GhNAPi transgenic lines in *Arabidopsis thaliana.*

Figure S4. Analysis of GhNAP-complemented lines dark treated for 5 d.

Figure S5. Effects of dark treatment for 3 d on detached leaves of GhNAP overexpressors.

Figure S6. Physiological and expression patterns of detached leaves of GhNAPi, *atnap*, and Col-0 lines under a dark environment for 5 d.

Figure S7. Phenotype and molecular analysis of the GhNAPi transgenic line in cotton.

Figure S8. Changes in content of MDA and soluble protein, and SOD and POD activity of the cotton leaf at the designed times in wild-type and GhNAPi lines.

Figure S9. Effects of dark treatment for 3 d on leaf discs of the wild-typeand GhNAPi lines.

Figure S10. Expression of ABA-related genes in wild-type and GhNAPi lines.

Table S1. Primers used for expression analysis by qRT-PCR.

Table S2. Primers used for constructing different vectors.

Table S3. Primers used for isolating the corresponding sequence.

Supplementary Data
