# Supplementary material for: A novel NAP member GhNAP is involved in leaf senescence in Gossypium hirsutum
Source: J Exp Bot. 2015 May 18;66(15):4669–82. doi: 10.1093/jxb/erv240 (PMC4507772; doi:10.1093/jxb/erv240)
Supplement: Supplementary Data [file supp_66_15_4669__index.html]

A novel NAP member GhNAP is involved in leaf senescence in Gossypium hirsutum — Supplementary Data 

# A novel NAP member GhNAP is involved in leaf senescence in *Gossypium hirsutum*

## Supplementary Data

Data files

- Supplementary Data - Supplementary Data
