# Supplementary material for: A novel NAP member GhNAP is involved in leaf senescence in Gossypium hirsutum
Source: J Exp Bot. 2015 May 18;66(15):4669–82. doi: 10.1093/jxb/erv240 (PMC4507772; doi:10.1093/jxb/erv240)
Supplement: Supplementary Data [file supp_erv240_148460Supplementary_Material.pdf]

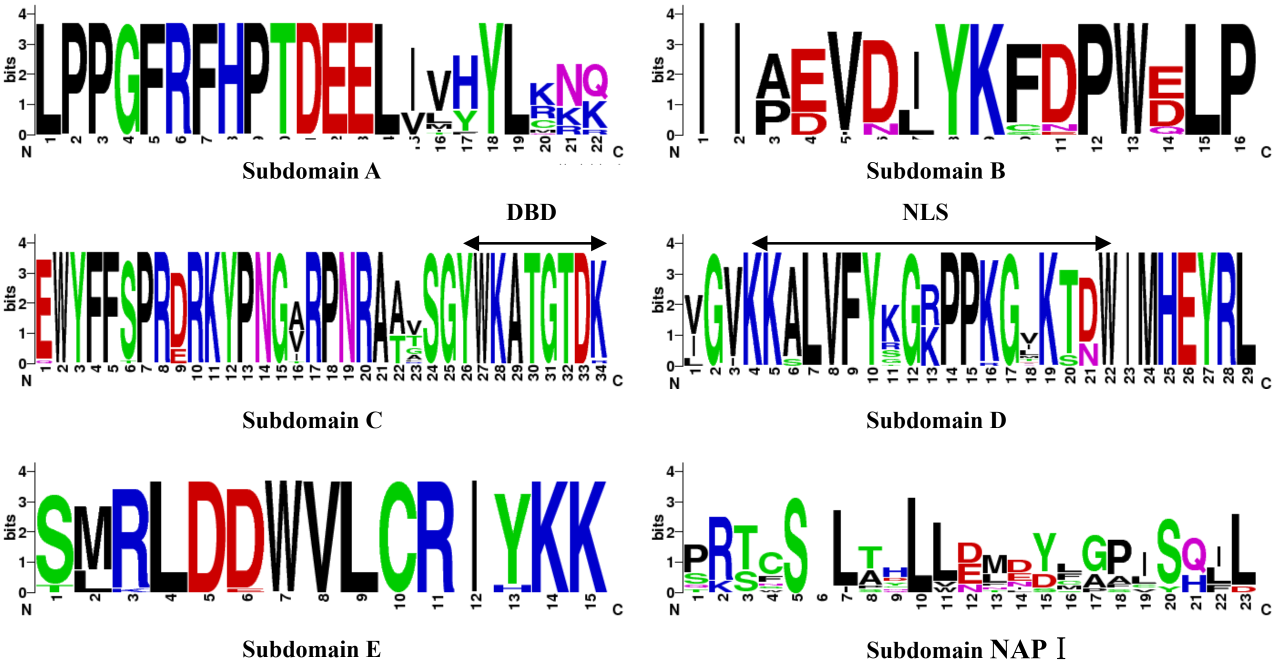

Fig. S1. The conserved domain of GhNAP protein. Sequence logos of NAC domain and a novel subdomain using WebLogo program. The height of letter designating the amino acid residue at each position represents the degree of conservation. The DBD domain on the top of subdomain C is thought to be involved in the DNA binding, and the NLS in the subdomain D stands for nuclear localization signal. The numbers on the x-axis represent the sequence positions in its corresponding conserved domains. The y-axis represents the information content measured in bits.

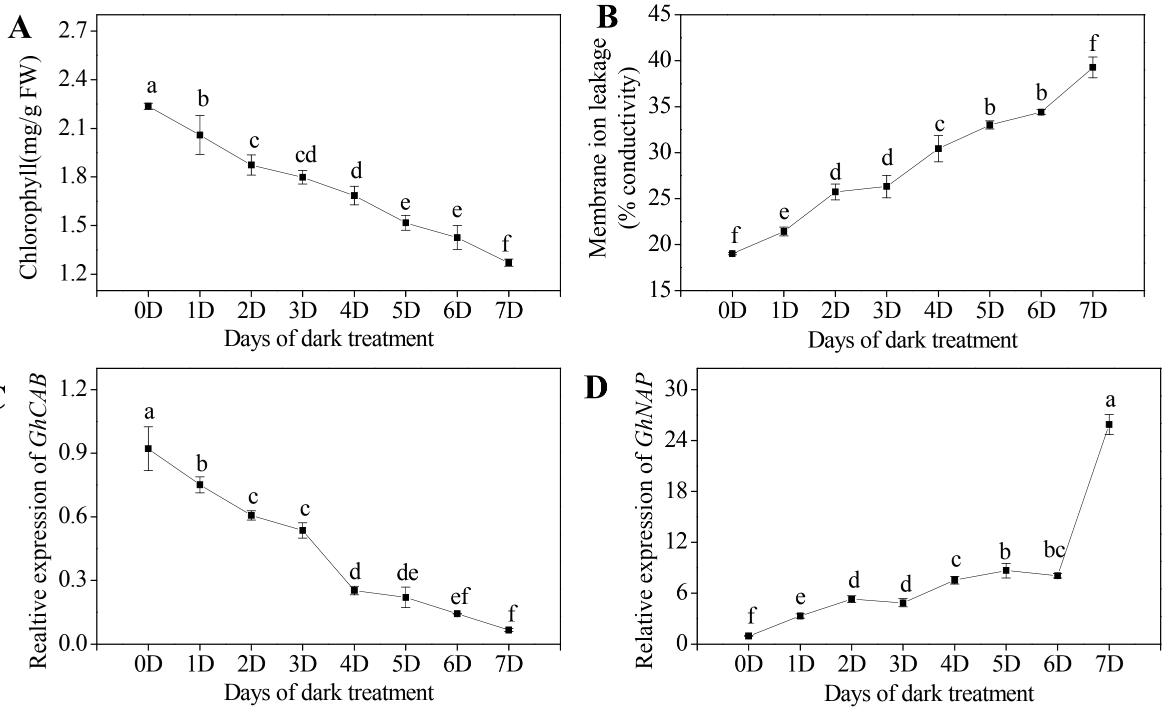

Fig. S2. Physiological and expression analysis of *GhNAP* in cotton leaves during extended darkness. Chlorophyll content (A), membrane ion leakage (B), relative expression of *GhCAB* (C) and *GhNAP* (D) induced by dark treatment for seven days in the cotton leaves.

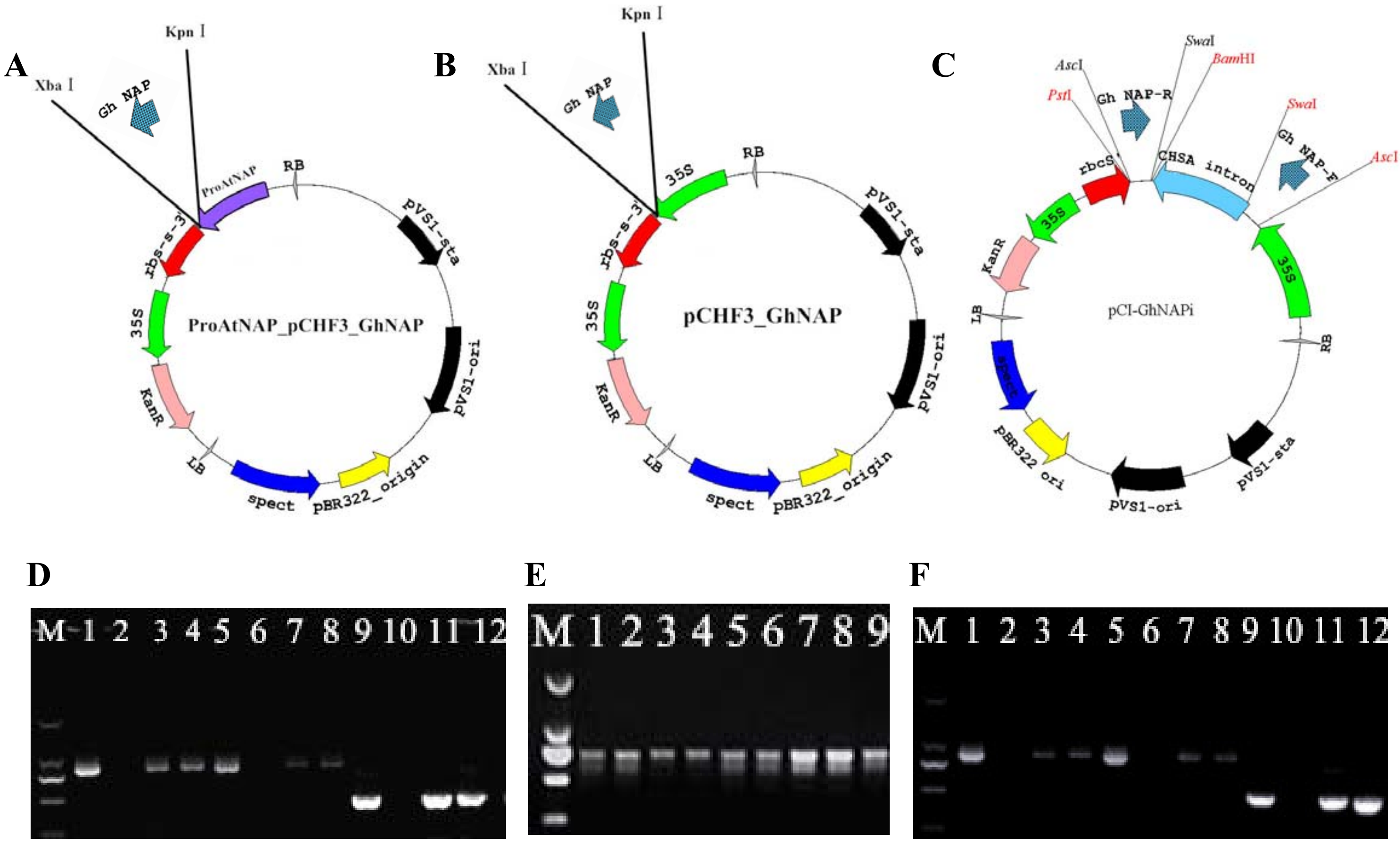

Fig. S3. Molecular analysis of the GhNAP_RE, GhNAP and GhNAPi transgenic lines in *Arabidopsis thaliana*. (A-C) Basal map for binary plasmid ProAtNAP_pCHF3_GhNAP (A), pCHF3_GhNAP (B) and pCI-GhNAPi (C) used for transformation. (D) PCR products to identify transgenic lines. Lane M, DNA marker DL 2,000 (TaKaRa); lane 1,5 and 9, positive control; lane 2, 6 and 10, non-transgenic Col-0; lane 3 and 4, GhNAP_RE transgenic lines; lane 7 and 8, GhNAP transgenic lines, line 11 and 12, GhNAPi transgenic lines. (E-F) RT-PCR analysis of the transgenic lines. RNAs were extracted from non-transgenic Col-0 and transgenic lines. *AtActin2* gene (E) was used as a standard control. Lane M, DNA marker DL 2,000 (TaKaRa); lane 1,4 and 7, non-transgenic Col-0; lane 2 and 3, GhNAP_RE transgenic lines; lane 5 and 6, GhNAP transgenic lines, line 8 and 9, GhNAPi transgenic lines. RT-PCR analysis of GhNAP_RE, GhNAP and GhNAPi cassette expression (F) in the transgenic lines. The sequence is the same as the Fig. S3D.

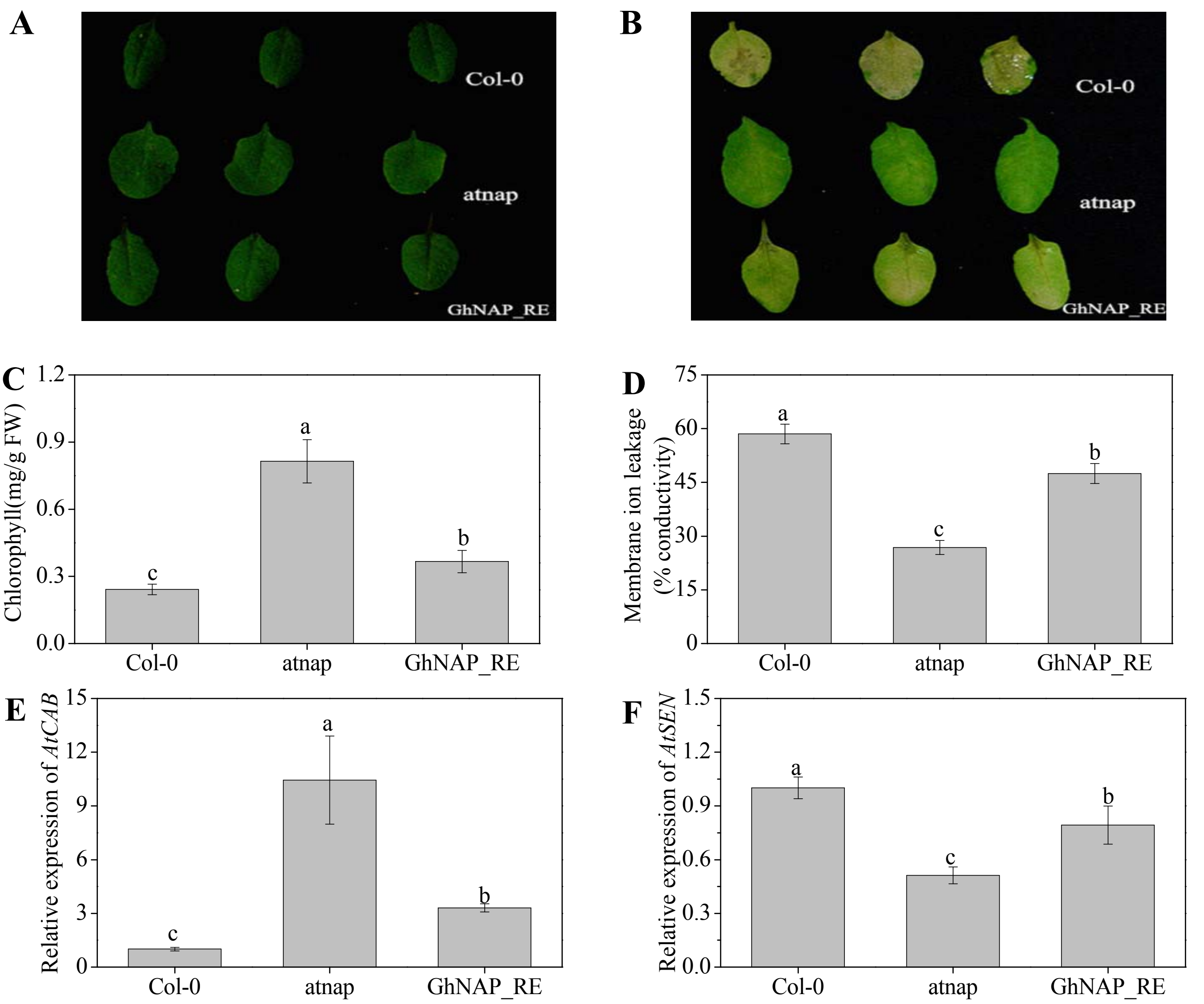

Fig. S4. Analysis of dark-treated GhNAP complemented lines for five days. (A, B) Phenotypes of detached leaves of GhNAP_RE, *atnap* and Col-0 lines treated by dark treatment for zero day (A) and five days (B). (C-F) Chlorophyll content (C), membrane ion leakage (D), relative expression of *AtCAB* (E) and *AtSEN* (F) in the dark treatment for five days.

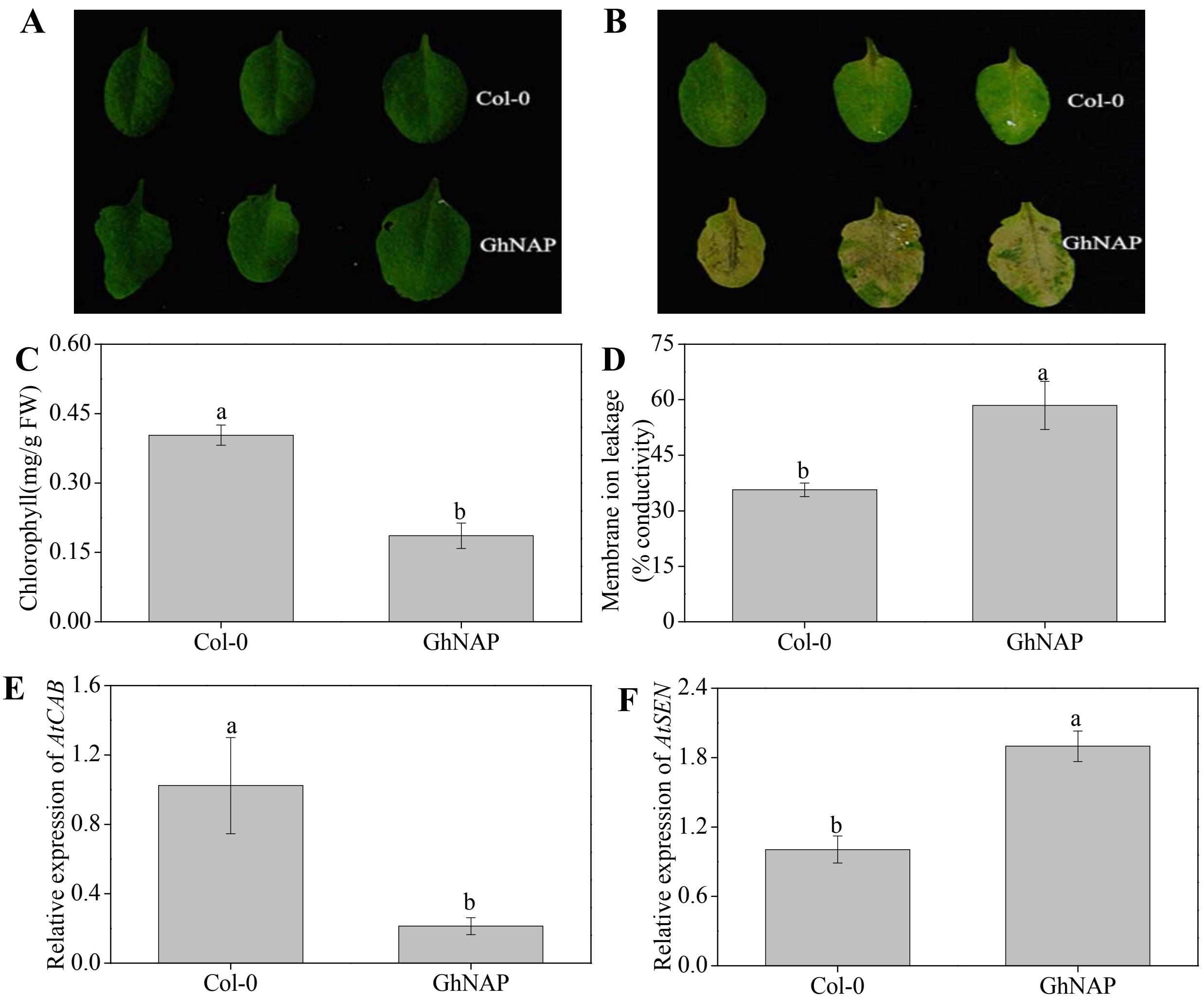

Fig. S5. Effects of dark treatment on detached leaves of GhNAP overexpressors for three days. (A, B) Phenotypes of detached leaves of GhNAP transgenic line and Col-0 treated by dark for zero day (A) and three days (B). (C-F) Chlorophyll content (C), membrane ion leakage (D), relative expression of *AtCAB* (E) and *AtSEN* (F) in the dark treatment for three days.

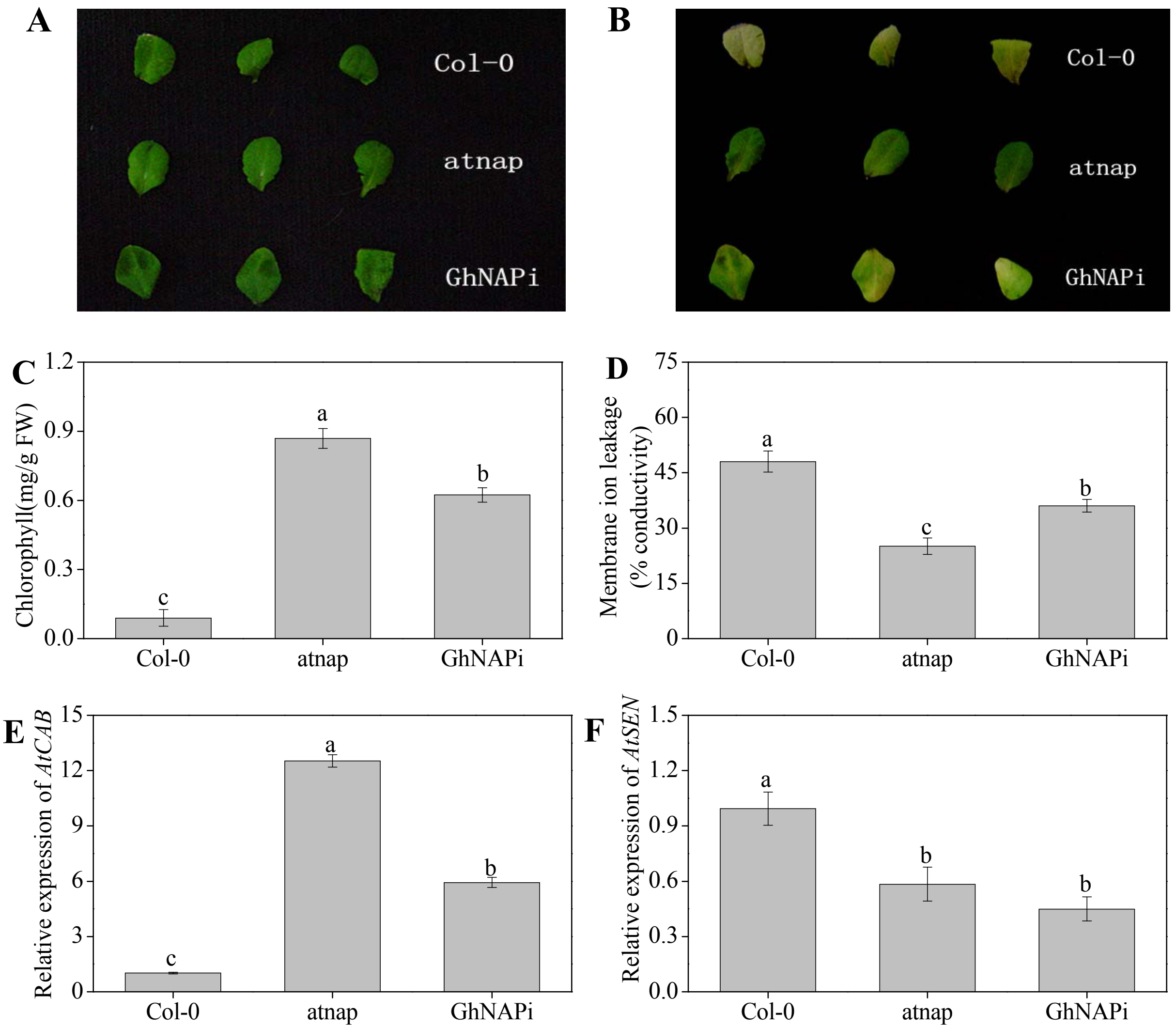

Fig. S6. Physiological and expression patterns of detached leaves of GhNAPi, *atnap* and Col-0 lines under dark environment for five days. (A, B) Phenotypes of detached leaves of GhNAPi, Col-0 and *atnap* lines incubated by dark for zero day (A) and five days (B). (C-G) Chlorophyll content (C), membrane ion leakage (D), relative expression of *AtCAB* (E) and *AtSEN* (F) in the dark treatment for five days.

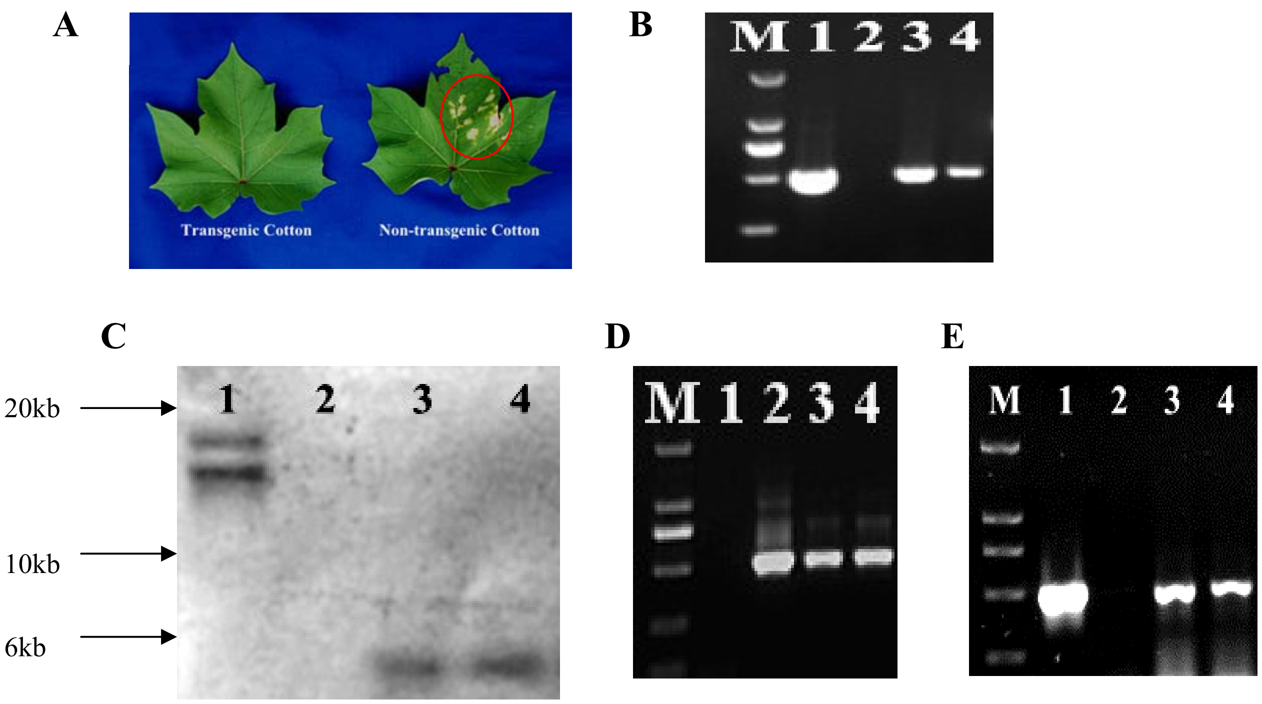

Fig. S7. Phenotype and molecular analysis of GhNAPi transgenic line (GhNAPi) in cotton. (A) Transgenic and non-transgenic seedling leaf scribbled with 500mg/L kanamycin. The red circle indicates non-transgenic leaf with the withered dot. (B) PCR products to identify the transgenic line. Lane M, DNA marker DL 2,000 (TaKaRa); lane 1, positive control (plasmid pCI-GhNAPi vector); lane 2, non-transgenic cotton; lane 3 and 4, GhNAPi transgenic line (including a replicate with independent DNA). (C) Southern blotting analysis for transgenic cotton. The cotton genome was digested with *Eco*RI and hybridized with probe after blotting onto membrane. Probe was labeled from PCR product of pCI-GhNAPi vector. Lane 1, positive control (plasmid pCI-GhNAPi vector); lane 2, non-transgenic cotton; lane 3 and 4, GhNAPi transgenic line (including a replicate with independent DNA). (D-E) RT-PCR analysis of GhNAPi transgenic lines. *EF1α* gene (D) was used as a standard control. Lane M, DNA marker DL 2,000 (TaKaRa); lane 1, blank control; lane 2, non-transgenic cotton; lane 3 and 4, GhNAPi transgenic line (including a replicate with independent RNA); RT-PCR analysis of GhNAPi cassette expression (E) in the transgenic line. The sequence is the same as the Fig. S7B.

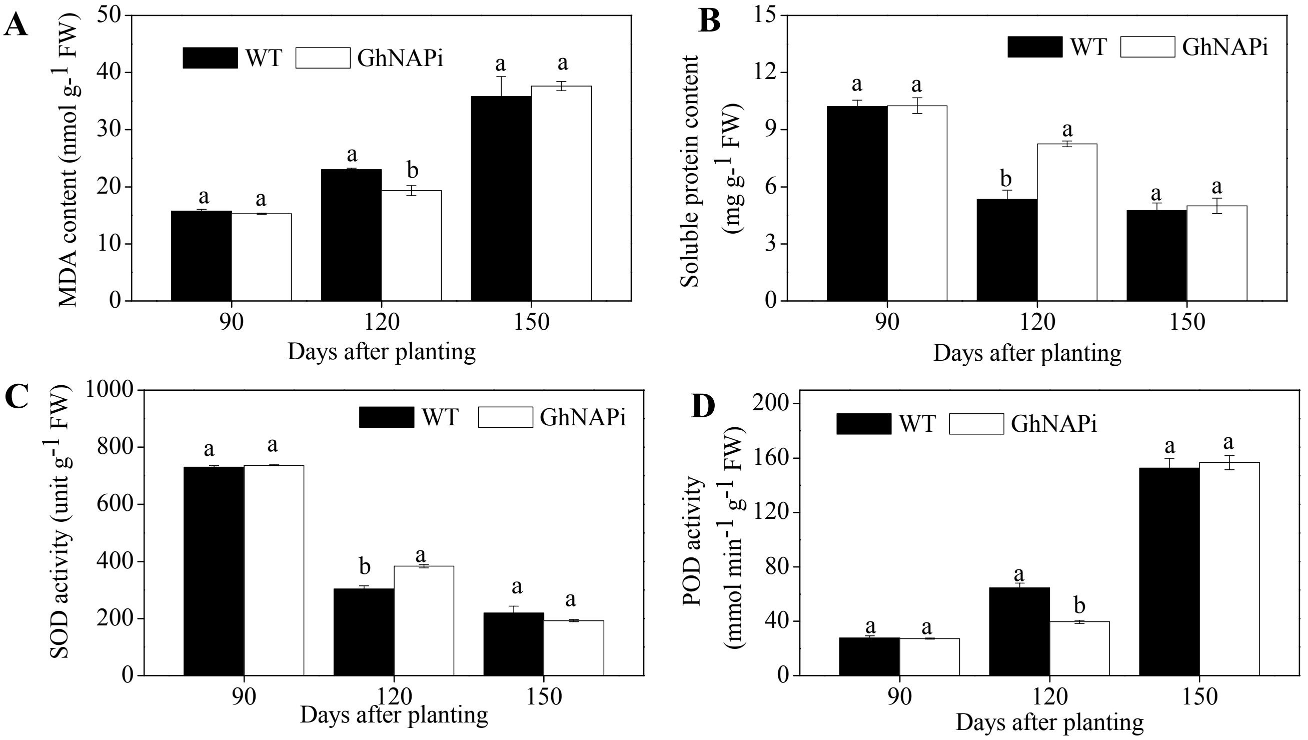

Fig. S8. Changes in content of MDA (A) and soluble protein (B), and SOD (C) and POD (D) activity of the cotton leaf at the designed time in WT and GhNAPi lines.

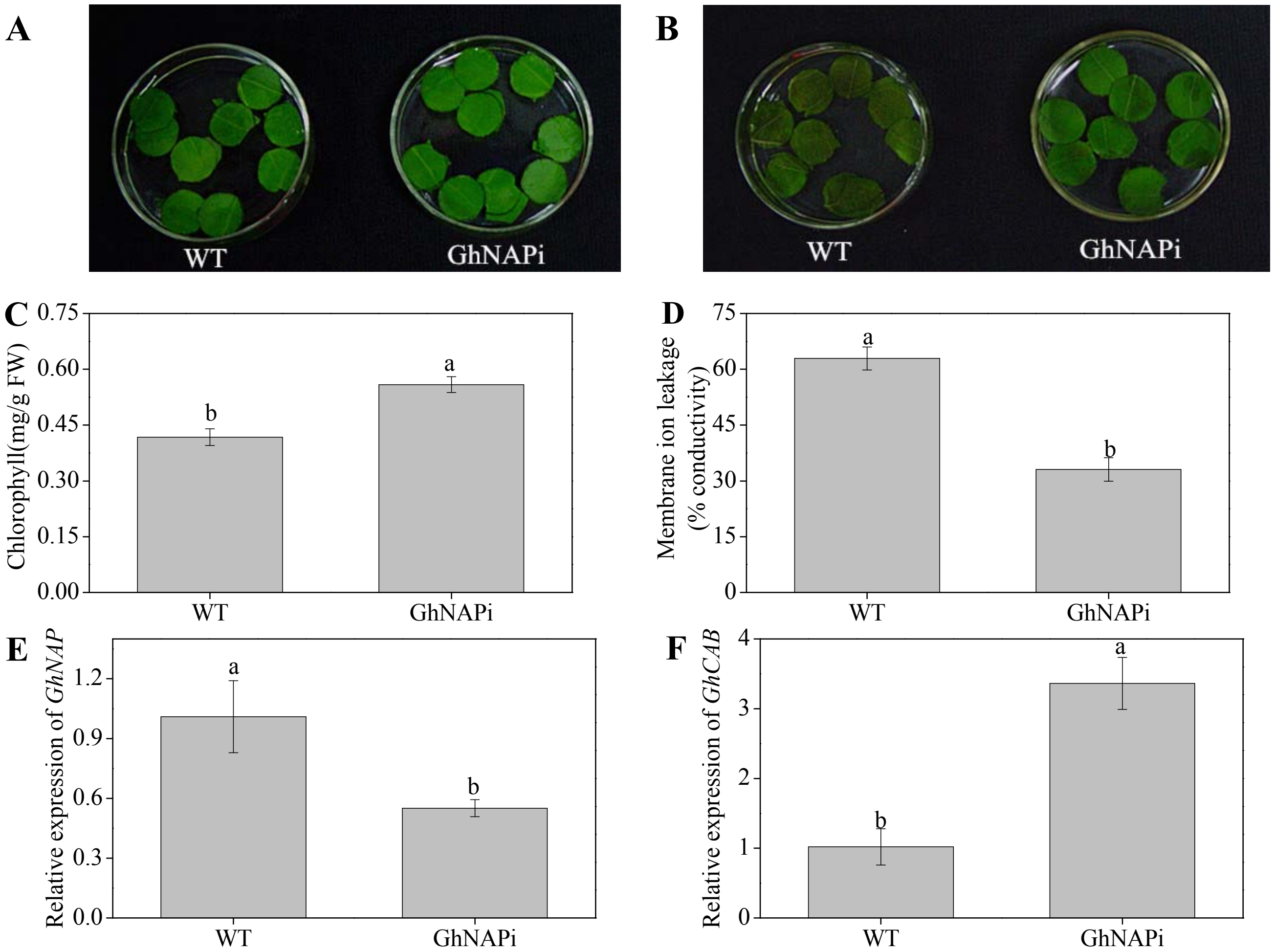

Fig. S9. Effects of dark treatment on leaf discs of the wild type (WT) and GhNAPi lines for three days. (A, B) Phenotypes of detached leaves of the wild type (WT) and GhNAPi lines incubated by dark for zero day (A) and three days (B). (C-F) Chlorophyll content (C), membrane ion leakage (D), relative expression of *GhNAP* (E), and G*hCAB* (F) in the dark treatment for three days.

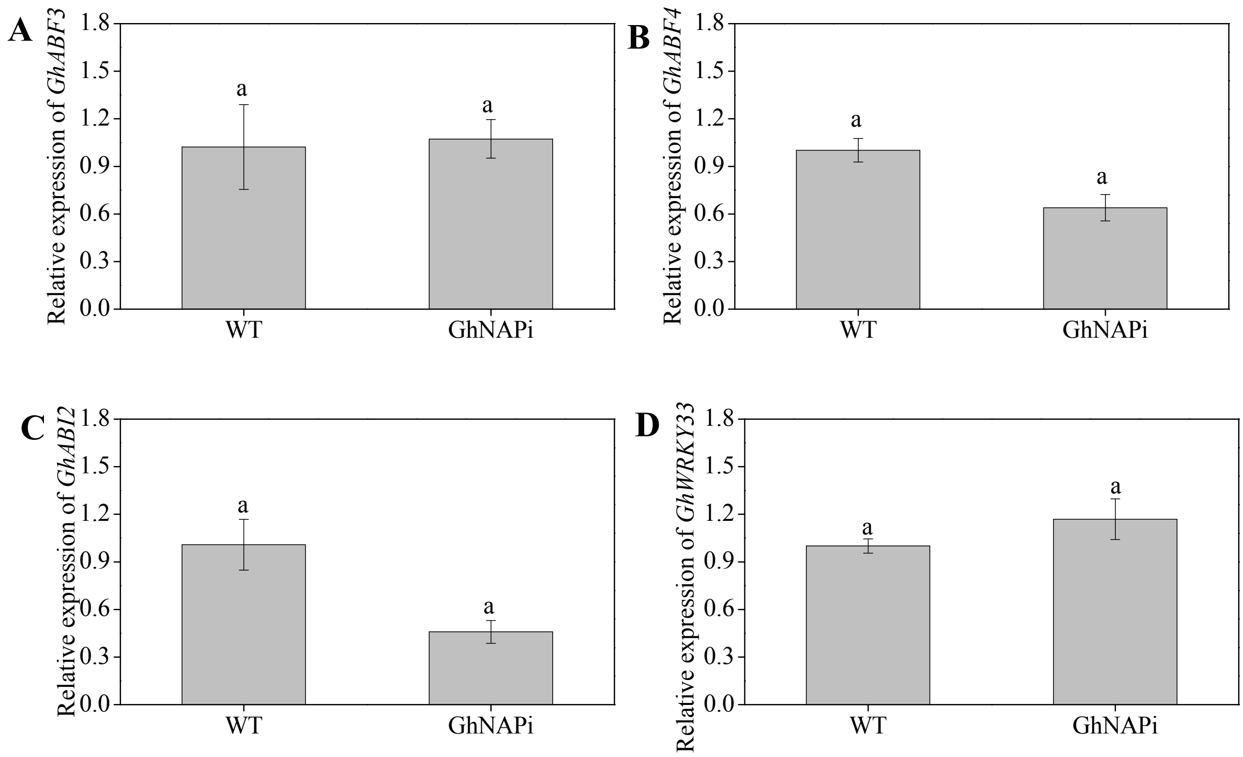

Fig. S10. Expression of ABA-related genes in WT and GhNAPi lines.

Table S1. *Primers used for expression analysis by qRT-PCR.*

Primer name	Sequence (5'→3')	Gene
qGhNAP-F	GCCCCAATTCACATGACACAGT	*GhNAP*
qGhNAP-R	TCTCAACATGGTCACCTGTGGT	*GhNAP*
qGhCAB-F	TGTCCCCGGAAAATGAACAAC	*GhCAB*
qGhCAB-R	TATGTGCTGCAGAAAATCATGCT	*GhCAB*
qEF1α-F	AGACCACCAAGTACTACTGCAC	*EF1α*
qEF1α-R	CCACCAATCTTGTACACATCC	*EF1α*
qGhSAG113-F	AAGACGCCGTCGCTATTCAT	*GhSAG113*
qGhSAG113-R	CACCTCATCGCCACATGAGA	*GhSAG113*
qGhMYC2-F	CCAGCCCTCCGTTTCTCTCCTCAGT	*GhMYC2*
qGhMYC2-R	CGAGCACGGTAGTGGCGGAGTAATC	*GhMYC2*
qGhMYB2-F	CGTTAACAGCAAGCAATTCAGGGAC	*GhMYB2*
qGhMYB2-R	GTAGGTAGTGGTGGTGGAGGAGGAT	*GhMYB2*
qGhABF3-F	GAATAGTTGGTTTACCATCTCCTGC	*GhABF3*
qGhABF3-R	AACTACTTTCTCCAATGCTGCACTG	*GhABF3*
qGhABF4-F	CCAGTCTTTCCAGAGCAGCA	*GhABF4*
qGhABF4-R	TCCCATCCCACCACTCTGAA	*GhABF4*
qGhWRKY33-F	GGAGTGAAAACCCACGCAGTTATTA	*GhWRKY33*
qGhWRKY33-R	TAGATTGAGGCTTGGGATGGTTATG	*GhWRKY33*
qGhABI2-F	AGTCAAATCCTCAAAGCCGAAAGTG	*GhABI2*
qGhABI2-R	ACAATAGTTCGCAACCTGACACCCT	*GhABI2*
qAtNAP-F	AGCCATTCACAGCGGTTCA	*AtNAP*
qAtNAP-R	GCTTACTTGCTCCTCTCTTCTTG	*AtNAP*
qAtSAG12-F	TGGATACGGCGAATCTACTAACG	*AtSAG12*
qAtSAG12-R	GCTTTCATGGCAAGACCACATAG	*AtSAG12*
qAtCAB-F	CCAGAGGCATTCGCTGAGTTG	*AtCAB*
qAtCAB-R	CCTTACCAGTGACGATGGCTTG	*AtCAB*
qAtSEN-F	GTCATCGGCTATTTCTCCACCT	*AtSEN*
qAtSEN-R	GTTGTCGTTGCTTTCCTCCATC	*AtSEN*
qAtActin2-F	CGCTCTTTCTTTCCAAGCTC	*AtActin2*
qAtActin2-R	AACAGCCCTGGGAGCATC	*AtActin2*

Table S2. *Primers used for constructing different vectors.*

Primer name	Sequence (5'→3')	Destination Vector
GFP-F	GCTCTAGAATGAGTAAAGGAGAAGAAC	pCHF3- GFP
GFP-R	GCTGCAGTCATTTGTATAGTTCATC	pCHF3- GFP
GhNAP-GFP-F	GGGGGGTACCATGGAAACAAAAACCAGC	pCHF3- GFP-GhNAP
GhNAP-GFP-R	GGGGTCTAGACTGAAATTGATACAGCAT	pCHF3- GFP-GhNAP
GhNAP-N-F	CGCCATATGATGGAAACAAAAACCAGC	pGBKT7-GhNAP-N
GhNAP-N-R	CCGGAATTCCTTCTTCTTGTAGATTCT	pGBKT7-GhNAP-N
GhNAP-C-F	CGCCATATGAACCCAGGGGGAAGAGGT	pGBKT7-GhNAP-C
GhNAP-C-R	CCGGAATTCTTACTGAAATTGATACAGCAT	pGBKT7-GhNAP-C
GhNAP-F-F	CGCCATATGATGGAAACAAAAACCAGC	pGBKT7-GhNAP
GhNAP-F-R	CCGGAATTCTTACTGAAATTGATACAGCAT	pGBKT7-GhNAP
AtNAPPro-F	CGGAATTCCATCCTAATCCTCATA	ProAtNAP- pCHF3
AtNAPPro-R	CCATGAGCTCCAGACAATTTAGAAAAC	ProAtNAP- pCHF3
GhNAP-CDS-F	GGGGGGTACCATGGAAACAAAAACCAGC	pCHF3-GhNAP
GhNAP-CDS-R	GGGGTCTAGATTACTGAAATTGATACAG	pCHF3-GhNAP
pCI-GhNAPi-F	GCTGCTGCAGGGCGCGCCGTGGAAGAATCAAATGC	pCI-GhNAPi
pCI-GhNAPi-R	CGGGATCCATTTAAATTCCCTGAATCAGTGTATTG	pCI-GhNAPi
GhNAPi-F	TGGAGAGGACACGCTCGAGT	pCI-GhNAPi transgenic text
GhNAPi-R	CCTTCCCACAATTCGTCGG	pCI-GhNAPi transgenic text
Y1GhNAP-F	GCCATGGAGGCCAGTGAATTCATGGAAACAAAAACCAGCTCTGAC	pGADT7-GhNAP
Y1GhNAP-R	CAGCTCGAGCTCGATGGATCCTTACTGAAATTGATACAGCATGGG	pGADT7-GhNAP
Y1GhSAG113_Pro-F	GAAAAGCTTGAATTCGAGCTCGAGGTTAGATCGGTAGCGCCAAAT	pAbAi-GhSAG113
Y1GhSAG113_Pro-R	AGCACATGCCTCGAGGTCGACAACAAGAACTAGAACCACACCATT	pAbAi-GhSAG113

Table S3. *Primers used for isolating the corresponding sequence.*

Primer name	Sequence (5'→3')	Gene
GhNAP-F	GAGGAATTCATGGAAACAAAAACCAGCTCTGAC	GhNAP
GhNAP-R	GCGTCGACTTACTGAAATTGATACAGCATGGGA	GhNAP
GhNAP-SP1	TCTTGAGTCCCTTTGGTGGCTTGC	GhNAP_Promoter
GhNAP-SP2	CCGTAGCCTTCCAATACCCTGACA	GhNAP_Promoter
GhNAP-SP3	CCATTCCTTTTCACCAAACTCTGC	GhNAP_Promoter
GhSAG113-SP1	CACAGTCCAGAGAAAATGATGCTC	GhSAG113_Promoter
GhSAG113-SP2	AATCTCCATCCTACGCCTCCTTGC	GhSAG113_Promoter
GhSAG113-SP3	CGCCATAACAAGAACTAGAACCACAC	GhSAG113_Promoter
